# Patterns of chemotherapy use and outcomes in advanced non-small cell lung cancer by age in England: A retrospective analysis of the population-based Systemic Anti-Cancer Treatment (SACT) dataset

**DOI:** 10.1016/j.jgo.2023.101581

**Published:** 2023-07-06

**Authors:** Sophie Pilleron, Eva J.A. Morris, David Dodwell, Kevin N. Franks

**Affiliations:** a Big Data Institute, Nuffield Department of Population Health University of Oxford, Oxford, United Kingdom; b Nuffield Department of Population Health, University of Oxford, United Kingdom; c Leeds Teaching Hospitals NHS Trust/ University of Leeds, Department of Clinical Oncology, Leeds, United Kingdom; d Leeds Institute of Medical Research at St James’s, School of Medicine, University of Leeds, United Kingdom

**Keywords:** Non-small cell lung cancer, Epidemiology, Survival, Population-based, Geriatric oncology

## Abstract

**Introduction:**

We described the patterns of chemotherapy use and outcomes in patients diagnosed with stage III or IV non-small cell lung cancer (NSCLC) by age in England.

**Materials and Methods:**

In this retrospective population-based study, we included 20,716 (62% stage IV) patients with NSCLC diagnosed from 2014 to 2017 treated with chemotherapy. We used the Systemic Anti-Cancer Treatment (SACT) dataset to describe changes in treatment plan and estimated 30 and 90-day mortality rates and median, 6-, and 12-month overall survival (OS) using Kaplan Meier estimator for patients aged <75 and ≥ 75 by stage. Using flexible hazard regression models we assessed the impact of age, stage, treatment intent (stage III), and performance status on survival.

**Results:**

Patients aged ≥75 years were less likely to receive two or more regimens, more likely to have their treatment modified because of comorbidities and their doses reduced compared to younger patients. However, early mortality rates and overall survival were similar across ages, apart from the oldest patients with stage III disease.

**Discussion:**

This observational study demonstrates that age is associated with treatment patterns in an older population with advanced NSCLC in England. Although this reflects a pre-immunotherapy period, given the median age of NSCLC patients and increasingly older population, these results suggest older patients (>75 yrs) may benefit from more intense treatments.

## 1 Introduction

Non-small cell lung cancer (NSCLC) represents about 85% of new lung cancer diagnoses, affecting about 20,500 people in England annually [1]. Over half of patients with primary NSCLC are diagnosed at an advanced stage [1]. Net survival is low; in 2010–2014 at one year from diagnosis survival was estimated at 44% (all stages and ages combined) in England and only 22% at three years [2].

Before the routine use of immunotherapy from 2017, cytotoxic chemotherapy was the main treatment for advanced NSCLC management for patients with good performance status [3].

As a result of the underrepresentation of older adults in randomized clinical trials [4], physicians do not have clear evidence on the benefit-risk balance in this population. Older adults commonly have poorer health status and fitness, more concomitant conditions, age-related physiological changes, and greater support needs compared to their younger counterparts [5]. Information on outcomes after chemotherapy in older patients with advanced NSCLC and the impact of chemotherapy on mortality at population level is scarce.

The Systemic Anti-Cancer Treatment (SACT) dataset includes data on systemic therapy in all patients diagnosed with cancer in England since 2014 [6].

Using the SACT dataset, we described the patterns of chemotherapy use and associated outcomes in patients diagnosed with stage III or IV NSCLC from 2014 to 2017 in England in relation to age.

## 2 Methods

### 2.1 Study Design and Data Sources

We conducted a retrospective observational population-based study using Systemic Anti-Cancer Treatment (SACT) dataset linked to the National Cancer Registration and Analysis Service (NCRAS) data.

The SACT dataset is a clinically led population-based resource that includes data on systemic therapy (excluding supportive treatment) delivered in secondary or tertiary settings for all patients (including children) diagnosed with cancer in England since 2014 [6]. Recording SACT data is mandatory for all NHS trusts in England. The dataset includes data on patient (e.g., age, sex), tumour (e.g., site, morphology, stage), regimen (e.g., treatment intent, type, date, weight and height, performance status, treatment adjusted because of comorbidity), cycles (e.g., date), drug details (e.g., dose, routes of administration), and outcomes (e.g., regimen modification). At the time of the analysis, only data up to 2017 were available. More details are available in Bright et al [6]

The NCRAS data are cancer registration data covering all of England. They include demographic information on patients and detailed cancer data (e.g., site, morphology, stage at diagnosis). Office National Statistics provide mortality data to NCRAS.

### 2.2 Study Participants

We included all patients diagnosed with stage III and IV NSCLC (ICD-10 codes: C34; morphology codes: see supplemental material) between January 1, 2014 and December 31, 2017 aged 18 or over, recorded in the SACT dataset as having received at least one dose of cytotoxic chemotherapy for lung cancer (see Supplemental Table 1), regardless of treatment intent. In case of multiple NSCLC diagnoses in a patient, we kept the first diagnosis in the analysis. All patients were followed for vital status up to December 31, 2018.

### 2.3 Chemotherapy Data

**Fig. 1:**
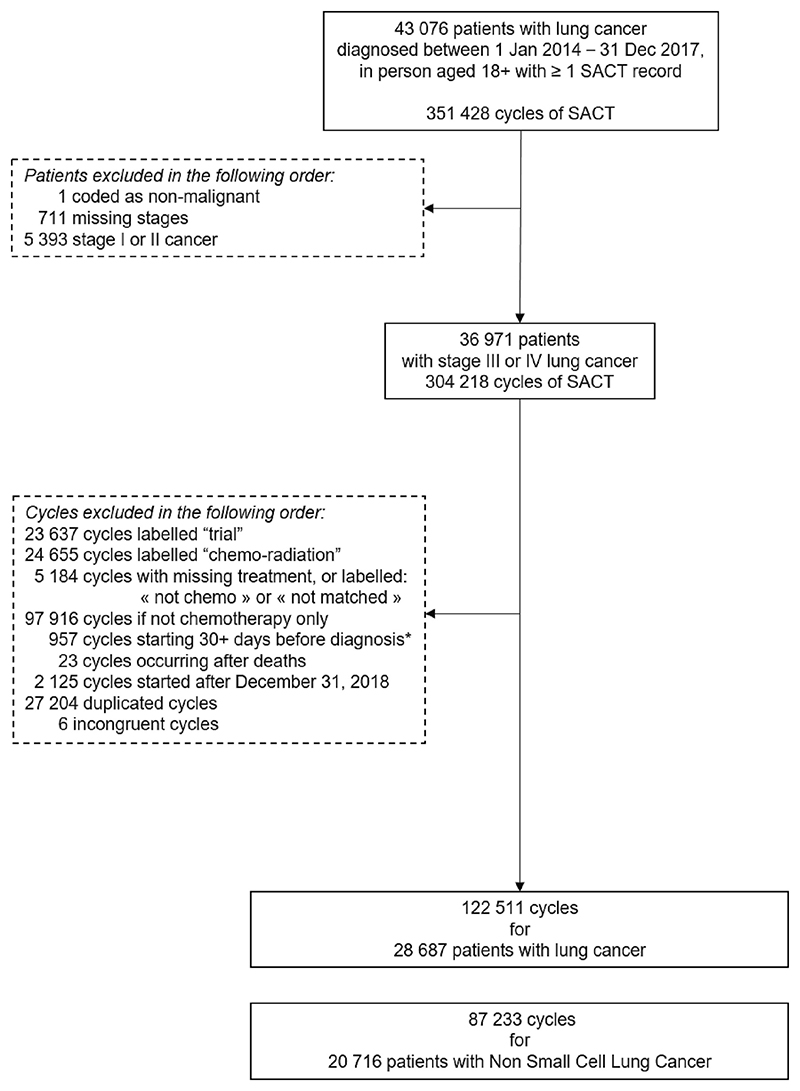
Flow chart.

All cycles of chemotherapy received after this date were excluded from analysis. We excluded cycles which were recorded as “trial,” “not chemo,” “not matched,” or “missing treatment group,” and duplicate records (Fig. 1).

We analysed regimen data including start date, treatment intent, treatment given, whether the regimen was adapted based on comorbidities, dose modification, or changes in treatment interval. Treatment intent was recoded into three categories: curative (including neo-adjuvant and adjuvant treatment), palliative, or disease modification (i. e., intent of controlling the disease without the expectation of eradicating it [7]). A cancer clinician checked for inconsistencies between chemotherapy regimen and treatment intent. In case of inconsistency or missing data, intent was recoded based on review of the regimens used in the context of NSCLC management (Supplemental Table 2) and stage. If a chemotherapy regimen may be used in both palliative and curative intent, we kept the original coding. If the original coding was “disease modification”, we kept as is. In stage IV NSCLC, if intent was coded as curative, it was recoded as palliative.

We also analysed the following SACT variables related to treatment adaptation [6]:

- Dose reduction = This identifies any regimens modified by reducing the dose of any anti-cancer drug administered at any point in the regimen after its commencement.
- Time delay = This identifies any regimens modified by extending the time between administration dates at any point in the regimen after its commencement.
- Regimen stopped early = This identifies any regimens modified by reducing the administration days below the number originally planned.

For each patient, we kept information regarding the first regimen they received. We also created a variable to indicate whether patients who received a first regimen with curative intent received chemotherapy with palliative intent subsequently.

We also used the start date of cycle of chemotherapy [6] and we calculated the median number of cycles each patient received.

In our analysis, we kept the performance status recorded at the start of the first chemotherapy cycle and recoded it into 0,1, 2+, and missing. This categorisation was based on NICE guidelines that, at the time, recommended palliative chemotherapy only for patients with a performance score of 0 or 1 [3].

### 2.4 Other Data

We retrieved characteristics of patients (age, sex, ethnicity) and tumours (morphology, stage at diagnosis) from NCRAS. Ethnicity is initially coded into the following categories: White British, White Irish, Any other White; Indian, Pakistani, Bangladeshi, Chinese, Other Asian; Black Caribbean, Black African, Other Black; White & Black Caribbean, White & Black African, White & Asian, Any other Mixed; and Other ethnic group. Because of small numbers, we dichotomized the ethnicity variable into White (White British, White Irish, Any other White) and non-White (all other ethnic categories).

### 2.5 Statistical Analysis

All analyses were stratified by stage. Percentages and medians with their interquartile ranges (IQR) were used to describe categorical and continuous variables (age, number of cycles), respectively.

We estimated mortality rates within 30 days of the first chemotherapy administration, that is an established governance metric in England [8,9]. We used the Kaplan Meier estimator to estimate median, 6-, and 12-month overall survival (OS) from the start of the first chemotherapy cycle in patients under 75 and those aged 75 or older. The age cut-off was chosen because evidence showed that patients with NSCLC aged 75+ were more likely to be undertreated than younger age groups [10,11].

To better describe how age at diagnosis influenced OS over the first year, we derived OS from the estimation of individual mortality hazard using a flexible hazard regression model. We modelled the hazard function as the exponential of B-spline of degree 3 with a knot located at the median of the distribution of survival times in patients who died. We included age as a B-spline of degree 3 with a knot at the median of the distribution of age in the whole sample. We ran models including age and treatment intent of the first regimen (for stage III only), and separately, models including age and performance status. Due to the descriptive nature of the study, no statistical tests were performed.

We performed statistical analyses using R statistical software (version 3.4.0; R Development Core Team, 2017) and we used the R ‘mexhaz’ package to model hazard and estimate survival [12].

### 2.6 Ethics

North of Scotland Research Ethics Service gave ethical approval for this work (REC/19/NS/0057).

## 3 Results

Out of 43,076 patients with lung cancer aged 18+ diagnosed between January 1, 2014 and December 31, 2017, we retained 7,884 stage III and 12,832 stage IV diagnosed with NSCLC (44% females in each stage) (Fig. 1).

Age at diagnosis was similarly distributed across stages, with comparable median age (68 [IQR: 62–73] in stage III and 67 [IQR: 60–73] years old in stage IV). Overall, 19.5% and 17.8% patients with stage III and IV NSCLC, respectively, were 75 years or older.

Table 1 presents characteristics of patients under and over 75 years old by stage at diagnosis. There were fewer females than males in older than younger patients in both stages (∼38% vs 45%). Ethnicity was equally distributed across age groups in both stages (>90% White). Older patients had poorer performance scores than their younger peers regardless of stage. Older patients with stage III disease were more likely to receive curative treatment than their younger peers (28.6% vs 21.6%, respectively). The median number of cycles received was 4 in all groups except for older patients with stage IV disease (median number = 3).

### 3.1 Changes in Treatment Plan

Older patients were more likely to have their treatment adjusted because of the presence of comorbidities (24% vs 18%) and to have reduced doses of chemotherapy than younger patients (36% vs 27%) at both stages of disease (see Table 2).

Thirty percent of patients with stage III NSCLC had delayed treatment regardless of age. However, treatment delay was more frequently recorded in older adults with stage IV disease compared to younger patients (32% vs 28%). About 18–19% of patients had their treatment stopped earlier than planned regardless of age and stage.

Older adults with NSCLC were less likely to have ≥2 regimens reported than younger adults, regardless of stage (42–44% vs 49%). Among patients who received ≥1 regimen, the percentage of those for whom a change in treatment plan was recorded was not noticeably different between age groups or stages. Older patients were more likely to have reduced doses of chemotherapy than younger patients (34% in patients under 75 years old in both stages vs 43% and 40% in older patients with stage III and IV, respectively).

### 3.2 Early Mortality and Overall Survival

Thirty-day mortality rates were ∼ 3% in patients with stage III disease, and 5–6% in those with stage IV disease with no age-related differences within stage (Table 3).

In respect to overall survival, older patients with stage III disease had a lower 6- and 12-month overall survival by 3.4%-points and 6.6%-points, respectively, and a shortened median survival by three months compared to patients ≤75 years of age. However, we did not observe age-related differences in 6- or 12-month OS (60% and 33%, respectively) or median survival (∼8 months in both age groups) in patients with stage IV NSCLC.

**Fig. 2:**
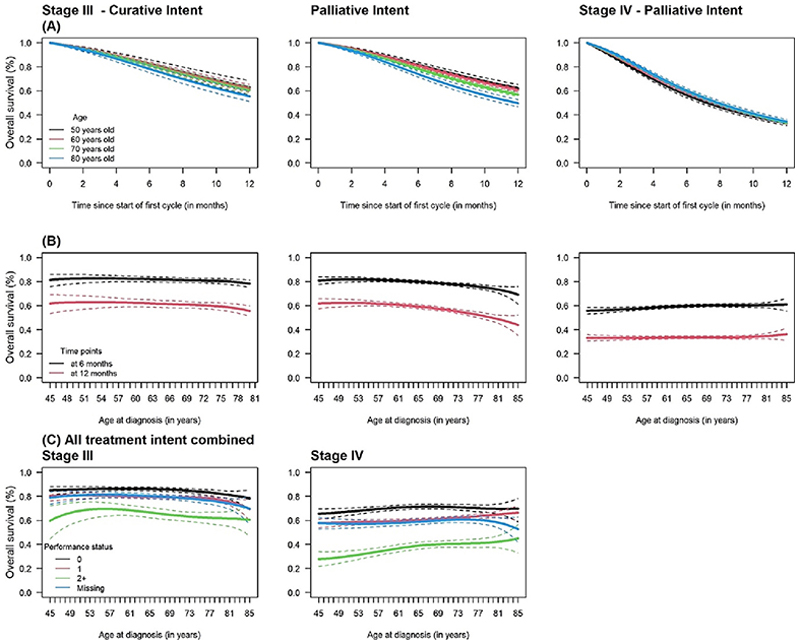
Overall survival (OS) over the initial year of the initiation of chemotherapy at ages 50, 60, 70, and 80; (B) 6 and 12-month OS by age at diagnosis, and (C) 6-month OS by age and performance status in patients diagnosed with stage III and IV non-small cell lung cancer in 2014–2017 and treated with chemotherapy (Note. Solid lines represent overall survival estimates and dashed lines represent 95% confidence intervals).

Figure 2 shows OS over the initial year after the start of the first chemotherapy cycle by age, stage, and treatment intent (Fig. 2 A and B), and performance status (Fig. 2 C). In stage III NSCLC, overall survival decreased over the first year and as age increased regardless of treatment intent. In stage IV NSCLC, there was no difference in overall survival across ages. There was also no age-related difference in 6- and 12-month overall survival (Fig. 2 B) except in patients with stage III disease receiving palliative treatment in whom survival gradually decreased from the age of 65.

Finally, 6-month overall survival did not differ between patients with a performance score of 0 or 1 but was poorer in those with score ≥ 2 in both stage III and IV (Fig. 2 C). Overall survival estimates in patients with missing performance status were in between those with score of 0/1 and those with score ≥ 2.

## 4 Discussion

This population-based study is the first to describe the use of chemotherapy and associated outcomes in a cohort of patients with NSCLC treated with chemotherapy in England by age before the introduction of immunotherapy. We showed chemotherapy was more likely to be modified in older patients than their younger peers, and older patients were less likely to have more than one regimen. However, age-related differences in early mortality and overall survival were marginal, notably in stage IV NSCLC, suggesting appropriate selection of patients for treatment.

Although our findings relate to the period before the introduction of immunotherapy in England, they are still relevant. National guidelines for the use of immunotherapy, combination immunotherapy and chemotherapy, and targeted therapy recommend that a patient have a performance status of 0–1 and not have a contra-indication for immunotherapy. In addition, due to the higher proportion of smoking-related lung cancer, the rates of patients suitable for targeted therapies are low, ranging from <1% to ∼10% in non-squamous non-small cell lung cancer [13]. Therefore, these results provide information on anticipated outcomes for the significant number of patients with lung cancer who are ineligible for, or unable to access, targeted therapy or immunotherapy. There are a few observational studies that describe the use of chemotherapy in patients with NSCLC covering more or less the same preimmunotherapy period. However, most of them were single institution studies and did not stratify by treatment intent or age, making comparisons difficult [14–18]. Moreover, some estimated survival from diagnosis, precluding comparison with our findings [14]. A study conducted in Leeds in England reported survival estimates stratified by histology and stage and showed an improvement in survival in stage IIIA and III-B-IV NSCLC, mainly in non-squamous cell carcinoma, between 2007 and 2012 and 2013–2017 [16]. However, results were not provided by age categories or by type of systemic therapy given. A US study reported a median survival of 9.7 months from the initiation of anticancer therapy in patients with stage IV NSCLC (median age = 67; 45% females) treated with chemotherapy or immunotherapy (∼20%), which is higher than in our study, but they did not report estimates by age [15].

Although there were more frequent modifications of treatment regimens in older patients, they had similar or somewhat better outcomes than younger patients. This finding suggests the selection of patients to receive chemotherapy was appropriate regardless of age. However, data were not available for the population who did not receive chemotherapy; this precludes any direct comparison. It would seem likely, however, that older patients who received chemotherapy were fitter than those who did not receive chemotherapy.

The main strength of the present study is the inclusion of all patients diagnosed with advanced NSCLC between 2014 and 2017 treated with chemotherapy in England, representing ∼22% of all patients with NSCLC diagnosed during the study period (based on an estimate of 82,000 NSCLC patients over the entire period [1]). Other strengths include good completeness of stage at diagnosis (about 2% missing stages) and long follow-up of patients. Our study has, however, limitations. Data (e.g., cycle number or regimen number) may be missing or incorrectly inputted [6] and characteristics such as comorbidity and geriatric conditions (e.g., frailty) for older patients were incomplete, precluding in-depth description of those patients who received chemotherapy. Finally, because of the better quality of the information on the date of cancer diagnosis, we used the age at diagnosis rather than the age at the treatment initiation in analysis. Because the delay between cancer diagnosis and the start of chemotherapy is generally short, we think that the impact on the findings presented will be negligible.

Given these limitations, it would be important to include an objective measure of clinical frailty, in addition to performance status, in future studies focused on older population to better guide which patients are able to safely receive systemic anti-cancer treatment and better tailor the treatment dose and intensity.

Some UK centres have already established a geriatric oncology service, such as the Senior Adult Oncology Service at The Christie NHS Foundation NHS Trust, Mancester, England (https://www.christie.nhs.uk/patients-and-visitors/services/senior-adult-oncology-service). The addition of comprehensive geriatric assessment and a multi-disciplinary approach with physiotherapy, dietetic, and occupational therapy input may improve the ability to deliver chemotherapy and new therapies (immunotherapy and molecular targeted agents) to this significant population of patients with lung cancer.

## 5 Conclusion

This national population-based study provides a snapshot of chemotherapy use and its associated outcomes in terms of early mortality and overall survival in patients with advanced NSCLC under and over 75 years old in England. Our results showed the absence of age-related differences in early mortality and survival. Our findings serve as baseline to monitor chemotherapy outcomes in both age groups, as new immunotherapy treatments for NSCLC have been made available since 2017.

## Supplementary Material

Appendix A- Supplementary data Supplementary data to this article can be found online at https://doi.org/10.1016/j.jgo.2023.101581.

## Figures and Tables

**Table 1:** Characteristics of patients diagnosed with non-small cell lung cancer in England in 2014–2017 treated with chemotherapy by stage and age group.

		Stage III			Stage IV	
		<75 years old	75+ years old		<75 years old	75+ years old
n		6349	1535		10,554	2288
Median age at diagnosis (years [IQR])		66.0 [60.0, 70.0]	77.0 [76.0, 80.0]		65.0 [58.0, 69.0]	78.0 [76.0, 80.0]
Females - n (%)		2856 (45.0)	589 (38.4)		4794 (45.5)	863 (37.7)
Ethnicity - n (%)						
White		5962 (93.9)	1447 (94.3)		9603 (91.1)	2161 (94.4)
Non-White		241 (3.8)	60 (3.9)		611 (5.8)	83 (3.6)
Unknown/Unstated		146 (2.3)	28 (1.8)		330 (3.1)	44 (1.9)
Performance status at start of the 1st cycle - n (%)						
0		1645 (25.9)	320 (20.8)		2338 (22.2)	370 (16.2)
1		2870 (45.2)	716 (46.6)		4647 (44.1)	1089 (47.6)
2+		540 (8.5)	196 (12.8)		1437 (13.6)	377 (16.5)
Missing		1294 (20.4)	303 (19.7)		2122 (20.1)	452 (19.8)
Treatment intent of the 1st regimen- n (%)						
Curative		1370 (21.6)	439 (28.6)		–	–
Palliative		4917 (77.4)	1078 (70.2)		10,477 (99.4)	2270 (99.2)
Disease modification		62 (1.0)	18 (1.2)		67 (0.6)	18 (0.8)
Change from curative to palliative intent among those who received curative treatment for their 1st regimen - n (%)		838 (61.2)	244 (55.6)		–	–
Median number of cycles recorded [IQR]		4.0 [2.0, 4.0]	4.0 [2.0, 4.0]		4.0 [2.0, 5.0]	3.0 [2.0, 4.0]

IQR: Interquartile range.

**Table 2:** Changes in chemotherapy treatment plans by stage at diagnosis and age group.

		Stage III			Stage IV	
		*<75*	≥75		<75	≥75
***First regimen recorded***						
Adjusted for comorbidity - n (% of non-missing)		904 (18.6)	286 (24.3)		1494 (18.4)	432 (24.4)
*Missing – n (% total)*		*1477* *(23.3)*	*359* *(23.4)*		*2416* *(22.9)*	*514* *(22.5)*
Dose reduction - n (% of non-missing)		1371 (26.9)	433 (36.1)		2347 (27.7)	648 (35.9)
*Missing – n (% total)*		*1259* *(19.8)*	*335* *(21.8)*		*2074* *(19.7)*	*485* *(21.2)*
Time delay - n (% of non-missing)		1069 (30.4)	250 (30.1)		1668 (28.3)	412 (32.4)
*Missing – n (% total)*		*2837* *(44.7)*	*704* *(45.9)*		*4642 (44.0)*	*1015* *(44.4)*
Stopped early - n (% of non-missing)		882 (18.4)	215 (19.2)		1468 (18.4)	316 (18.7)
*Missing – n (% total)*		*1547* *(24.4)*	*416* *(27.1)*		*2580* *(24.5)*	*600* *(26.2)*
***In any subsequent regimens recorded***						
# patients with >1 regimen recorded		3119 (49.1)	681 (44.4)		5144 (48.8)	965 (42.2)
Adjustment for comorbidity - n (% of non-missing)		446 (19.3)	107 (21.2)		754 (19.4)	169 (23.3)
*Missing – n (% total)*		*805* *(25.8)*	*175* *(25.7)*		*1250* *(24.3)*	*241* *(24.9)*
Dose reduction (% of non-missing)		832 (33.9)	226 (43.0)		1430 (34.8)	298 (39.7)
*Missing – n (% total)*		*668* *(21.4)*	*156* *(22.9)*		*1030 (20.0)*	*215* *(22.3)*
Time delay - (% of non-missing)		684 (38.5)	140 (36.6)		1275 (41.1)	226 (40.0)
*Missing – n (% total)*		*1341 (43.0)*	*298* *(43.8)*		*2042* *(39.7)*	*400* *(41.5)*
Stopped early - (% of non-missing)		467 (20.5)	86 (18.0)		914 (23.8)	151 (21.8)
*Missing – n (% total)*		*839* *(26.9)*	*204 (30.0)*		*1311* *(25.5)*	*271* *(28.1)*

**Table 3:** 30-day mortality rates, 6-, and 12-month and median overall survival from the start of the first cycle of chemotherapy in patients with non-small cell lung cancer by stage at diagnosis and age group.

		Stage III						Stage IV				
		<75			≥75			<75			≥75	
		# deaths	% [95%CI]		# deaths	% [95%CI]		# deaths	% [95%CI]		# deaths	% [95%CI]
30-day mortality rate		169	2.7 [2.6–2.7]		48	3.1 [3.0–3.2]		653	6.2 [6.1–6.3]		117	5.1 [5.0–5.2]
6-month overall survival		1242	80.4 [79.4–81.4]		353	77.0 [74.9–79.1]		4240	59.7 [58.7–60.6]		903	60.4 [58.4–62.5]
12-month overall survival		2554	59.4 [58.2–60.6]		721	52.8 [50.3–55.4]		6989	33.2 [32.3–34.1]		1514	33.4 [31.5–35.4]
Median survival (months)		4232	15.8 [15.2–16.4]		1122	13.0 [12.2–13.8]		9174	7.7 [7.5–7.9]		2010	7.9 [7.5–8.2]

CI: confidence interval.

## Data Availability

De-personalised study data may be made available on request to accredited researchers who submit a proposal that is approved by the PHE Office for Data Release.
